# Family Assistance Experiences of Adolescents in Marriage Immigrant Families

**DOI:** 10.3390/children12070862

**Published:** 2025-06-30

**Authors:** Yeseul Jeong, Kyung-Sook Bang

**Affiliations:** 1 College of Nursing, Keimyung University, Daegu 42601, Republic of Korea; yeseuljeong@kmu.ac.kr; 2 Research Institute of Nursing Science, Keimyung University, Daegu 42601, Republic of Korea; 3 College of Nursing, The Research Institute of Nursing Science, Seoul National University, Seoul 03080, Republic of Korea

**Keywords:** adolescent, immigrant family, family assistance, parentification, qualitative research, phenomenology

## Abstract

**Background/Objectives:** Family assistance by adolescents (e.g., cooking, cleaning, listening to family members) is a common phenomenon. However, the impact of such assistance on adolescent development remains a topic of debate. Increasingly, the importance of adolescents’ own perceptions and interpretations is being emphasized in understanding how family assistance influences their development. Adolescents in marriage immigrant families may face unique psychosocial challenges as they support their immigrant parents. This study explores the family assistance experiences of adolescents in marriage immigrant families. **Methods**: Data were collected through one-on-one in-depth interviews with 10 adolescents aged 13–18 years from currently married marriage immigrant families, all of whom were born in Korea. The interviews were conducted between October and November 2022. The data were analyzed using interpretative phenomenological analysis. **Results**: The family assistance experiences were categorized into three main themes, namely, “Old enough to help—naturally, and rightly so”, “Foreign mother whom I naturally come to help”, and “Unavoidable family assistance, even under pressure”, and 10 subordinate themes. The adolescents provided support naturally, grounded in familial obligation and empathy toward their immigrant mothers. When the native Korean father shared the responsibilities, the assistance was not perceived as burdensome. However, diminished paternal involvement, traditional gender role expectations, and unmet emotional or academic needs led to more negative perceptions and psychological stress. **Conclusions**: Support systems are needed to ensure that adolescents do not assume sole responsibility for both the native father’s and the immigrant mother’s roles within marriage immigrant families.

## 1. Introduction

In South Korea, international marriages have rapidly increased from 3.7% of all marriages in 2000 to 10.1% in 2023 [1]. Accordingly, the number of children from multicultural families has increased approximately fivefold, from 58,007 in 2008 to 299,440 in 2022 [2]. In particular, the number of adolescent children aged 13 to 18 surged from 6176 in 2008 to 80,840 in 2022 [2].

Adolescence is a developmental period marked by extensive physical, psychosocial, and cognitive growth, during which individuals prepare for a healthy transition into adulthood. During this time, various factors can influence adolescents’ growth and development, and family support is one such factor. Family assistance refers to activities such as helping with household chores or caring for younger siblings, as well as providing moderate emotional support, including listening to and empathizing with family members [3]. This concept is distinct from not only parentification, whereby developmentally inappropriate adult roles or responsibilities are expected from children [4,5], but also young caregiving, whereby children provide intensive and time-consuming care such as assisting with meals, dressing, or toileting for ill, disabled, or aging family members [3]. During adolescence, individuals experience significant physical growth, social interactions, skill acquisition, and enhanced cognitive and social awareness, all of which can improve their capacity to assist others [6]. Consequently, adolescents may become more capable of supporting their families, and parents may, in turn, come to expect more assistance from them. Performing newly assigned roles and meeting these expectations can, in turn, influence adolescents’ development and well-being.

Family assistance has both positive and negative effects on adolescents’ health and development [3,7]. For example, positive health outcomes, such as improved sleep quality, fewer physical symptoms, and reduced physiological stress responses, have been reported [8,9,10]. Conversely, other studies have found associations between family assistance and negative outcomes, such as increased substance use or elevated levels of C-reactive protein (CRP), a biomarker of inflammation [11,12]. While the fragmented nature of the existing studies presents a limitation, the inconsistency in the findings highlights the complexity of the effects that family assistance may have on individuals, suggesting that its impact is not straightforward.

For adolescents from immigrant families, the types and levels of family assistance expected may differ due to their family’s migration background; nonetheless, both positive and negative outcomes have been reported in this group as well. Immigrant adolescents may act as cultural and linguistic brokers for their parents—who typically adapt to the host society more slowly—and may take on responsibilities uncommon among their non-immigrant peers, such as translating official documents or assisting with family financial issues [13]. While such roles may expose these adolescents to experiences of discrimination or psychological stress due to excessive responsibilities [14,15], they may also foster family adaptation and the development of adolescents’ social skills, self-efficacy, and self-esteem [13].

These divergent findings in the existing literature suggest that the impact of family assistance is shaped not only by the task itself but also by contextual factors and adolescents’ own perceptions of their roles. One study reported that family assistance in high-conflict households is linked to increased substance use [11]. Additionally, Lam et al. [16] found that family assistance has been associated with depressive symptoms and declining academic performance only in contexts of high parental conflict or low family values [16]. Similarly, Lazarevic [15] found that among immigrant adolescents, negative family dynamics can exacerbate the burdens of discrimination and lead to role reversals, indicating the importance of context in shaping these experiences. These findings highlight the need to explore adolescents’ subjective experiences of family assistance.

Despite this need, the extant literature has focused on parentification or young caregiving [14,17], and comprehensive research on adolescents’ everyday family assistance and their own perceptions and meanings of such experiences is lacking.

Particularly, examining the family assistance experiences of adolescents from marriage immigrant families—who are likely to face compounded difficulties owing to their background—from their own perspectives is essential as a foundational step in supporting their healthy development and adjustment. Therefore, this study seeks to comprehensively explore the lived experiences and meanings of family assistance among adolescents from marriage immigrant families through interpretative phenomenological analysis (IPA).

## 2. Materials and Methods

### 2.1. Research Design

This study employed a qualitative research design using IPA to deeply examine the family assistance experiences of adolescents from marriage immigrant families.

### 2.2. Data Collection

#### 2.2.1. Participant Selection

This study recruited participants through purposive sampling. Participants were adolescents, aged 13–18 years (mean age: 14 years), who were born in Korea and from marriage immigrant families, in which the parents were currently married. All the participants could communicate in Korean, were legally minors, living in households, and without intellectual or physical disabilities. All the immigrant parents were mothers, and their countries of origin were the Philippines, Vietnam, Pakistan, and Indonesia.

#### 2.2.2. Participant Recruitment

Participants were recruited by posting study announcements at two multicultural centers, one community children’s center, and one online community for marriage immigrants. Adolescents who saw the announcement and expressed interest contacted the researcher directly, received a detailed explanation of this study, and subsequently decided to participate.

#### 2.2.3. Data Collection Procedure

Data were collected through one-on-one in-depth interviews conducted from November to December 2022. The interview method, location, and time were decided based on participant preferences. Nine interviews were conducted at cafes or a private room in the center, and one was conducted using a real-time video-conferencing platform. As the data collection occurred during the COVID-19 pandemic, the participant chose to participate online. The mode of interview did not pose any limitations.

To create a comfortable atmosphere, the researcher offered refreshments and engaged in icebreaking activities before the interviews. Participants also completed a general demographic questionnaire. Special care was taken to build a rapport with participants, particularly by the adult female researcher, who explicitly expressed appreciation and respect for the adolescents as experts on their own experiences, thereby minimizing the discomfort stemming from adult–adolescent hierarchies.

The interviews were guided by a semi-structured protocol developed through a pilot study. The interview guide consisted of approximately ten open-ended questions. Examples of these questions included the following: “Can you describe a typical day at home?”, “How is household labor shared in your family?”, “How do you usually help your family?”, “How do your family members respond when you help?”, and “What is your relationship like with your parents?” Follow-up and probing questions were used when necessary to further clarify or explore participants’ responses.

Each interview lasted for an average of 1 h and 35 min, and all the interviews were conducted once per participant. The interviews were audio-recorded, the researcher took field notes during the interviews, and all the recordings were transcribed verbatim by the researcher. Two participants reviewed their transcripts to verify the accuracy of the transcriptions and ensure that their statements were accurately reflected. The data collection was concluded when saturation was reached, that is, when no new themes or information emerged and the interview content became repetitive with no additional insights.

### 2.3. Data Analysis

The data were analyzed following the IPA approach outlined by Smith and Nizza [18] and Smith, Flowers, and Larkin [19]. IPA is a qualitative research methodology that inductively explores how individuals perceive and make sense of their lived experiences. Rooted in phenomenology, hermeneutics, and idiography, IPA involves interpreting data collected from participants through the processes of phenomenological reduction and the hermeneutic circle. This approach emphasizes an in-depth, rigorous, and detailed examination of specific cases, aiming to illuminate participants’ experiences through rich descriptions and analyses that capture both individual and shared meanings.

The data collection and analysis were conducted concurrently. We maintained a reflexive stance throughout the process to prevent the fore-conception from distorting participants’ lived experiences. The transcripts and field notes were repeatedly examined to not only become immersed in the data but also identify the distinctive and structural elements of each participant’s experience. Exploratory notes were developed and synthesized into experiential statements. These statements were then physically arranged and clustered to develop a table of personal experiential themes for each participant. After conducting the individual case analyses, a cross-case analysis was performed to derive group experiential themes and subthemes, which were subsequently labeled as themes and subthemes, respectively.

### 2.4. Ethical Considerations

This study was reviewed and approved by the Institutional Review Board (IRB) of Seoul National University to ensure the ethical protection of research participants (IRB No. 2209/003-012), and the ethical procedures were strictly followed.

Before the data collection, an online pre-meeting was conducted with potential participants to explain this study using a shared screen displaying the consent form and study explanation. Participants were informed of this study’s objectives, procedures, use of audio recordings, data anonymization, and data storage and usage. Participation was entirely voluntary, and it was explained that participants could withdraw at any time without any disadvantage; if withdrawn, all the data would be immediately discarded. Written informed consent was obtained from both the participants and their legal guardians, and consent was reconfirmed on the day of the interview before proceeding.

### 2.5. Rigor

The researchers were trained in qualitative research methods and had hands-on experience of conducting and analyzing qualitative studies. Prior to the data collection, the relevant literature and media were critically reviewed, and one researcher (YJ) reflected on the personal experiences of having immigrant relatives. A pilot study was conducted to develop the interview guide, which was further refined during this study based on the emerging themes. Purposeful sampling ensured the selection of appropriate participants, and the data collection continued until saturation was reached. Efforts were made to create a comfortable interview environment, empathize with participants’ experiences, and affirm them as experts on their own lived realities. The researcher (YJ) collected data directly from participants and transcribed it verbatim. To enhance the analytical rigor, the analysis was thoroughly interpretative and focused on individual cases before synthesizing across cases, following Smith and Nizza’s [18] approach. Bracketing was applied throughout the process to reduce the bias from the researchers’ fore-conceptions. Additionally, feedback was obtained from a third party experienced in qualitative research, and the results were member-checked with two participants.

To enhance transparency and rigor, this study followed the COREQ checklist [20], detailing the participant recruitment, data collection, analysis, and findings. Efforts were made to ensure a logical writing structure and clarity, with detailed descriptions and appropriate quotations.

## 3. Results

### 3.1. General Characteristics of the Participants

The study participants were 10 adolescents from marriage immigrant families. The majority were female (n = 7, 70.0%) and high school students (n = 6, 60.0%) (Table 1). The average age of the participants was 15 years (range: 13–18), and the average ages of their fathers and mothers were 53.6 years (range: 45–63) and 39.1 years (range: 34–55), respectively. The mothers’ countries of origin were the Philippines (n = 4, 40.0%), Vietnam (n = 4, 40.0%), Pakistan (n = 1, 10.0%), and Indonesia (n = 1, 10.0%). According to the adolescent participants, their immigrant mothers’ Korean language proficiency was rated as “high” in the following areas: speaking by eight participants (80.0%), listening by six participants (60.0%), reading by two participants (20.0%), and writing by two participants (20.0%).

### 3.2. Experiences of Family Assistance Among Adolescents from Marriage Immigrant Families

The family assistance experiences of adolescents from marriage immigrant families were categorized into three main themes, namely, “Old enough to help—naturally, and rightly so”, “Foreign mother whom I naturally come to help”, and “Unavoidable family assistance, even under pressure”, with a total of ten subthemes being identified (Table 2).

#### 3.2.1. Old Enough to Help—Naturally, and Rightly So

This theme describes the participants’ internal standards and perceptions that led them to accept family assistance as a natural and rightful responsibility.

##### Things That an Adolescent—Not a Child—Can Take on

All the participants engaged in various household chores, such as laundry and cleaning, to help their families. While some assistance was voluntary, most occurred in response to direct requests from parents. The adolescents recognized that the type and degree of assistance expected by their parents changed as they matured. Tasks that were once difficult owing to physical limitations or immaturity were now manageable thanks to their growth and development. They understood the new expectations as something feasible due to their physical and emotional maturity, which helped them accept these roles regardless of whether the tasks were voluntary.

*“The food waste bin is kind of big. So, my parents started asking me to take it out once I got tall enough”. (Participant 7)*

*“Now that I’m 14, I guess they trust me more. When I was younger, they didn’t really believe in me”. (Participant 1)*

The participants expressed satisfaction and a sense of achievement when completing difficult or higher-responsibility tasks. When their parents acknowledged or praised their efforts, they perceived themselves as capable and competent, thus contributing to a positive self-image.

*“My mom and dad say I have a knack for taking care of my little sibling. When the baby sleeps well, it feels nice and fulfilling”. (Participant 1)*

##### Learning and Preparing for the Tasks I Will Face When I Grow up

Most participants helped their mothers, who primarily managed the household duties. They learned chores step-by-step from their parents and assisted them accordingly. The responsibilities were not overly burdensome in terms of the difficulty, quantity, frequency, or responsibility, so the participants viewed the tasks as somewhat annoying but manageable. Moreover, they regarded the experience as a form of learning and preparation for adulthood—a way of acquiring skills they would eventually need.

*“When I vacuumed the wrong way, my dad said, ‘This is how you should do it’, and demonstrated it to me”. (Participant 8)*

*“I think of it as practice for when I grow up. I’ll have to clean like this then, too”. (Participant 3)*

##### Family Means Helping One Another

The participants frequently heard from both family and society that children should help their parents and that family members were supposed to support each other. This message helped internalize family assistance as a natural responsibility. Additionally, their own experiences of receiving help from relatives purely because of familial ties further reinforced the value of mutual support. To them, helping their family was a way to not only uphold family values but also repay the support they had received. The participants evaluated themselves as “good” people by duly embracing these family values, showing that family support can help form positive self-evaluation.

*“When I was sick, my mom and I went to the university hospital with my aunt. She helped with the diagnosis and explained everything to my mom”. (Participant 6)*

*“I might not be the best at everything, but I think I’m decent. I listen to my parents and try to do what they ask, even if I’m not good at it”. (Participant 1)*

#### 3.2.2. Foreign Mother Whom I Naturally Come to Help

This theme captures how the participants perceived their immigrant mothers as individuals in need of help and how assisting their mothers became a familiar, normalized part of their lives from a young age.

##### Being Told to Help My Immigrant Mother

The participants repeatedly reported hearing throughout their upbringing that they should help their immigrant mothers. Their Korean fathers would often explain that their mothers came to Korea alone, without family, and may feel lonely, encouraging the children to understand and support them. Moreover, their immigrant mothers often requested help because of physical or emotional fatigue resulting from working. These messages naturally shaped the perception that helping their lonely and exhausted mothers was expected and necessary, leading the participants to accept family assistance with little resistance. However, in situations where their own emotional struggles conflicted with their mothers’ needs, the participants sometimes felt compelled to suppress or relinquish their own needs. This reveals that the acceptance of family assistance is not always solely grounded in positive motivation.

*“(When I tell my dad I’m upset with mom) he says I should help her because she came from Vietnam all alone without her family and must be really lonely… When I hear that, I feel like I have to understand and help her, not that I want to”. (Participant 9)*

##### Driven by Empathy for My Immigrant Mom

By living closely with their immigrant mothers, the participants came to understand that their mothers had different backgrounds and experiences from those of themselves or native Koreans. Through everyday observations, they recognized the chronic difficulties and isolation that their mothers faced in Korean society. Sometimes, they tried to put themselves in their mothers’ shoes and empathized with the hardship of living in a foreign country. In particular, hearing about the discrimination their mothers faced simply for being immigrants reinforced their motivation to support them.

*“If I went to another country where I didn’t speak the language and went to the hospital with a friend, and people were speaking in words I didn’t understand… I’d ask my friend what was going on”. (Participant 5)*

*“When my mom talks about being discriminated against, I feel like it must be really tough for her. It makes me think, ‘I should be on her side at least.’” (Participant 10)*

##### Assisting with Mom’s Small but Constant Language Struggles

Language brokering for their immigrant mothers was an inevitable part of family assistance for all the participants. While their mothers could communicate reasonably well through speaking and listening in Korean, they still needed help with reading and writing. Although the whole family shared in supporting the mother’s language needs, the Korean father often played the primary role—handling official communications with schools, banking, and interpreting text messages. The participants also contributed to the language brokering, especially when their fathers were unavailable. While this task felt burdensome when they were younger, as their Korean language and problem-solving skills improved, the participants began to view it as a minor, manageable responsibility—“annoying, but not difficult”. Regardless of its difficulty, language brokering was a recurring duty for all the participants with immigrant mothers.

*“Mom isn’t great at figuring out Korean words on her own, so she always asks dad. If he’s not home, she asks me”. (Participant 9)*

*“For things like writing a report in Korean, I think I started doing that in first grade. It used to be hard, sitting there going over it and fixing it… But these days, I can just run it through a grammar checker quickly, so it’s okay”. (Participant 7)*

#### 3.2.3. Unavoidable Family Assistance, Even Under Pressure

This theme explores the experiences of adolescents from marriage immigrant families who provide assistance to their families while under psychological pressure.

##### Carrying the Weight of the Father’s Role Too Soon

In most participants’ households, the father was seen as the primary economic provider and symbolic head of the family. This perception persisted even after the father’s retirement, and he continued to be regarded as the pillar of the family. For the participants, helping their fathers was not limited to everyday support; it implied sharing major responsibilities, such as earning income and maintaining the family. Under normal circumstances, they were not expected to assume or share these roles. However, due to structural changes such as their father’s retirement in his 60s, deteriorating health, or the birth of a much younger sibling, the participants began to gradually take on some of their fathers’ responsibilities. In this context, they were expected to prepare for financial independence and internalize a sense of responsibility to eventually step into the father’s role in the near future.

*“When I was told we were having a new baby, my dad said, ‘I’m getting old, and if something happens to me, you—my eldest son—must lead the family. You can’t act like a child anymore. I’m sorry, but I want you to mature earlier than other kids.’” (Participant 8)*

In Korea’s college-entrance-focused society, it is atypical for children to immediately support themselves financially upon becoming adults. The participants also believed that studying hard was their main duty as adolescents. Thus, being asked to shoulder part of the father’s burden caused them to feel overwhelmed and confused. However, they understood that such requests reflected the family’s difficult circumstances and felt unable to reject them. The belief that they must share the weight of being the family’s pillar caused considerable psychological pressure, often leading to feelings of helplessness and depression.

*“The thought of having to earn money as soon as I become an adult is kind of stressful. So I try not to think about it… a sort of helplessness?” (Participant 4)*

*“Getting the role of the next family head at such a young age made me feel mentally overwhelmed and stressed. I didn’t believe in depression before, but now… I realize it’s real”. (Participant 8)*

##### Forced into Traditional but Outdated Roles

Some traditional values were used to justify forcing the participants to help their families, even when they did not agree with such values. The participants wished to reject outdated beliefs and obligations, but, as minors, they could not easily oppose adult authority within the household and were sometimes coerced into undesired family assistance. In particular, patriarchal gender role stereotypes had negative effects on all the adolescents, regardless of their gender. On the one hand, female participants were scolded if they were not good at cooking or cleaning, causing them to feel unjustly and unfairly treated. Male participants, on the other hand, internalized traditional norms that labeled them as “the next family head”, leading them to feel immense responsibility and pressure. These outdated gender role expectations contributed to feelings of inequality, negative emotion, and psychological burden among the participants.

*“Times have changed a lot, right? But I just think to myself, ‘It’s not like that anymore…’ When grandma says things like, ‘You have to eat what you’re given if you want to be liked by your future in-laws, and you must be good at housework if you want to get married.’ I hate that”. (Participant 7)*

*“If you’re the eldest son, the family’s pillar, you must be able to take responsibility, bear the burden, endure hardship… and I think you’re expected to have a strong sense of sacrifice too”. (Participant 8)*

##### Unassisted Academic Struggles

The participants viewed “studying hard” and “obeying their parents” as forms of helping their parents. Focusing on academics reduced the parents’ worries and was therefore considered a form of emotional support. Likewise, obeying parental instructions without causing stress was seen as a way to support the parents psychologically.

*“I think it’s good to be respectful to the parents who went through so much to have me. I try not to speak rudely, and I try to listen when they ask me to do something. I just want to keep them from being stressed”. (Participant 1)*

Despite their strong sense of duty toward pursuing academic success, many participants struggled due to a lack of parental support. According to the participants, immigrant mothers often did not attend school meetings or access academic information because of language and experiential limitations. Unlike typical households where the mothers actively support their children’s education, the immigrant mothers in these families are unable to provide substantial help with academics or career planning. Although the participants sometimes sought help from their fathers, they often could not provide effective support due to a lack of knowledge, limited availability, or lower educational attainment. While academic support is generally considered a parental responsibility, the participants had to accept and assist themselves in this role, leading to feelings of disappointment and burden. Some even had to take responsibility for younger siblings’ studies in place of their parents, which they found unfair and stressful.

*“Most people talk to their parents about GPA and the college entrance exam. But my mom and dad don’t really know much about that, so we don’t talk about it. I wish I could talk to them—even just to get some advice”. (Participant 10)*

*“It’s hard to ask my dad to help my younger brother with homework… Honestly, no one in our house is good at studying. My dad only finished high school, and my mom doesn’t speak Korean well. So if not me, then who?” (Participant 6)*

##### A Language Barrier Too Thick to Truly Touch the Heart

With the exception of a few participants who primarily communicated with their mothers in English (Participants 2, 3, and 4), most did not speak their mothers’ native language, and communication with their mothers occurred primarily in Korean. While the participants generally rated their mothers’ Korean language proficiency as “high”, this was found to be limited to basic, everyday communication. The participants experienced language barriers with their immigrant mothers particularly in situations requiring detailed explanations or emotional communication. Due to limitations in their Korean proficiency, some mothers employed a closed and directive communication style when asking for help, or gave vague instructions without sufficient explanation. These interactions were often perceived as authoritarian, leading to misunderstandings and negative emotions. Emotional and supportive conversations that might have resolved such issues could not take place effectively because of the language barrier. Consequently, the participants felt frustration and resentment toward their mothers, and repeated communication failures sometimes led them to develop negative perceptions vis-à-vis their mothers’ foreign identities.

*“Since she’s Vietnamese, she often talks in a very shortened way. It made me wish mom spoke better Korean… or even that she were just Korean. Then we could communicate better”. (Participant 9)*

*“Mom tends to just say, ‘Do what I say, I’m tired’, and things like that… Since she’s a foreigner, I think it’s hard for her to express things properly in Korean”. (Participant 7)*

## 4. Discussion

This study is a qualitative investigation conducted to explore the experiences and meanings of family assistance among adolescents from marriage immigrant families. In-depth interviews were conducted with 10 Korean-born adolescents from marriage immigrant families, and the data were analyzed using IPA.

The first theme, “*Old enough to help—naturally, and rightly so*”, revealed that adolescents from marriage immigrant families naturally engaged in helping their families as part of their development, grounded in strong family values. They perceived these responsibilities as part of growing up and a preparatory process for adulthood. This finding aligns with prior studies that emphasize the significance of family values in collectivist Asian cultures [14], where family values have been shown to predict adolescents’ family assistance behaviors [21]. Asian adolescents often grow up in environments that emphasize loyalty to family, obedience to parents, and expectations to contribute to family welfare. Even when faced with difficulties, such cultural contexts can foster the acceptance of assigned roles [14]. The participants in this study unreservedly accepting routine requests for family assistance as natural can be interpreted as being influenced by this cultural background.

The natural acceptance of family support roles observed among the participants in this study may stem not only from their cultural backgrounds but also from the adolescents’ positive evaluations of helping their families—such as feelings of pride, a sense of self-efficacy, and fulfillment of their roles as family members. Aumann and Titzmann [22] reported that adolescents from German immigrant families in Switzerland engaged in more technology mediation activities than their native peers. This finding is particularly noteworthy given that the study focused on European immigrant families, rather than Asian or Latin American families, who are typically more associated with strong familism values. Such results suggest that family assistance behaviors among immigrant adolescents may not solely be the result of internalized cultural values.

Previous studies have also found that immigrant adolescents’ support for their families is related to both the social adaptation of immigrant parents [23] and adolescents’ positive emotional states [10]. These findings imply that family support can be perceived not merely as a duty or cultural expectation but as a subjective and positive experience. The increase in positive affect observed during family assistance in Shen and colleague’s [10] study may be associated with adolescents’ sense of contributing to the family, fostering agency and a sense of positivity. Similarly, the participants in the present study expressed feelings of pride or evaluated themselves as “good” individuals through their family assistance experiences, suggesting that helping one’s family can be understood as a self-affirming and agentic act rather than a mere obligation. This aligns closely with the findings from previous research.

In cultural groups that emphasize family values, family assistance has been linked to positive developmental outcomes, including increased positive affect [10] and prosocial behaviors [9,21]. Thus, helping one’s family may serve not only as role fulfillment but also as a contributor to adolescents’ psychological and social development. However, when the expectations for adolescents to support their families become excessive, negative outcomes may arise. For instance, Mejia and colleagues [24] found that while fulfilling familial expectations was associated with a positive self-image among early Asian adults, failure to meet such expectations correlated with a negative self-image and symptoms of depression. Therefore, for family assistance to promote adolescent development, identifying and addressing value mismatches within the family, such as traditional gender roles, is necessary. Additionally, the level of expectations held by both adolescents and their families must be assessed. Moreover, where discrepancies exist, adjustments should be made accordingly.

The participants’ experiences of family assistance were twofold: a positive preparation for adulthood and a source of perceived unfairness. These experiences could vary depending on the degree of parental involvement. While household labor can function as a process of socialization that fosters responsibility and the internalization of family norms, it can also serve as a means of compensating for labor shortages caused by demographic or economic limitations [25]. When parents share household tasks with their children, this can promote family closeness, instill values around labor, and foster responsibility and patience [26]. Conversely, when children are left to perform such tasks alone, they may interpret their contributions as a form of forced labor, especially when seen as a substitute for adult responsibilities [27]. This perception of unfairness can lead to negative outcomes, such as depression, substance use, and diminished self-efficacy or self-esteem, and may be indicative of parentification [5].

These results particularly highlight the psychological vulnerabilities of female adolescents in relation to family assistance. Several studies have shown that daughters are more likely to take on family responsibilities compared with sons [7,28,29]. Moreover, some findings indicate that the amount of housework that daughters perform is comparable to that of their fathers [28]. However, few studies have directly examined the relationship between fathers’ participation in housework and daughters’ experiences of parentification or perceived unfairness. Therefore, future research should explore how fathers’ involvement in household labor affects female adolescents’ perceptions and mental health more specifically.

The second theme, “*Foreign mother whom I naturally come to help*”, illustrates how the participants form their perceptions of helping their immigrant mothers based on their upbringing and everyday experiences. Messages about their mothers’ hardships and isolation, conveyed over time, fostered empathy and a sense of moral obligation. The participants consistently acted as language brokers for their immigrant mothers, perceiving the role as a familial responsibility rather than a major burden. Difficult tasks were typically handled by the Korean—non-immigrant—fathers; thus, the adolescents did not bear the full burden. This contrasts sharply with previous research that suggests language brokering can lead to a reversal of parent–child roles and an intense psychological burden [14,30]. For example, Cho and colleagues [14] studied early adult children in Asian immigrant families in the U.S. who took on vital administrative tasks for survival, such as handling health insurance, housing applications, and utility bills, instead of their parents. These tasks were often accompanied with the fear of making costly errors and added significant stress. Conversely, the participants in this study grew up in South Korea and had native-born Korean fathers, who belonged to the majority of the population. These fathers handled official documents and public communications, reducing the children’s burden and creating a different language-brokering experience. Furthermore, considering previous research emphasizing the importance of language acquisition through formal schooling [31], it is likely that adolescents in multicultural families in South Korea—having been born and raised in the country and having learned Korean as a first language through the national curriculum—experienced little difficulty communicating in Korean on behalf of their mothers.

Furthermore, Crafter and Iqbal [32] emphasized that the context in which language brokering occurs is important, especially in hostile environments. Immigrant children acting as intermediaries with native adults may face discrimination and microaggression, reinforcing their marginalized identity and negatively affecting their well-being. They may also feel anxious about making mistakes because of their limited proficiency in either language [30]. However, the participants in this study were fluent in Korean and did not bear the sole responsibility for complex tasks, thereby significantly lowering their exposure to such risks. The supportive involvement of their Korean fathers allowed them to share the burden of brokering in a less stressful manner.

The theme “*Unavoidable family assistance, even under pressure*” revealed that the adolescents in marriage immigrant families often found themselves in inescapable roles, leading to psychological strain. The participants were burdened by having to assume their fathers’ responsibilities, forced to adhere to outdated traditional values, and left to manage their academic and emotional needs without adequate parental support, often leading to disappointment or even disillusionment with their marriage immigrant family background.

Despite the presence of socially vulnerable immigrant mothers, the family assistance provided by the adolescents in marriage immigrant families did not amount to full-scale parentification, such as the reversal of parent–child roles. A key reason for this was the presence and involvement of Korean, native-born, fathers. As members of the dominant cultural group, these fathers were capable of addressing the challenges faced by the immigrant mothers because of their familiarity with Korean society and its systems. Although the children were also born and raised in Korea, their limited experience and capabilities meant that they could not assume such responsibilities to the same extent as their fathers. When difficulties arose in helping their mothers, the adolescents were able to rely on their fathers to take over, which significantly reduced their psychological burden and limited the scope of their responsibilities. However, the heavy role of Korean fathers in these households also meant that any absence or loss of function—due to illness, aging, or retirement—posed a significant risk to the family. Some participants reported experiencing psychological and economic stress because of their fathers’ older age or deteriorating health. The average age of the participants’ fathers was 53.6 years, compared to 39.1 years for the mothers. This reflects national data from 2004 to 2008, when immigrant women commonly matched with significantly older Korean husbands [33]. For example, between 2004 and 2009, the age gaps between immigrant wives and their Korean husbands were as follows: 0–9 years (24.7–33.3%), 10–19 years (46.9–54.8%), and 20+ years (16.5–18.4%). This contrasts with Korean couples from the same period, where only 4.0–5.9% had an age gap of 10 years or more [34]. These differences suggest that marriage immigrant families with adolescent children today often have fathers who are significantly older than their peers in non-multicultural households. In this study, the participants with fathers near to or over 60 years of age reported changes in their households due to the father’s physical or economic limitations, resulting in psychological distress, such as pressure to become financially independent (Participant 4), anxiety about their father’s health (Participant 5), and a burden related to becoming the next family head (Participant 8). These findings highlight the socioeconomic and emotional vulnerabilities of children in marriage immigrant families with older fathers. There is a clear need for future research and policy development to assess and support the psychological well-being of these adolescents.

The participants in this study perceived “studying hard” and listening to their parents as forms of emotional support for their parents. This perception aligns with previous findings that report a positive association between adolescents’ family caregiving behaviors and their academic engagement, suggesting that in family-centered cultures, a sense of familial responsibility can be internalized as academic motivation [9]. However, emphasizing academic immersion in the absence of substantial emotional and academic support from parents may lead to heightened stress among adolescents.

In cultures influenced by Confucian values, children’s academic achievement is regarded as a crucial familial duty. Consequently, children are not expected to contribute much to household chores [35]. Furthermore, supporting their academic pursuits is greatly emphasized. Nevertheless, the participants in this study were often expected to assist both their mothers, who were raising children alone in unfamiliar environments, and their fathers, who supported them, placing them in dual caregiving roles.

Notably, children in marriage immigrant families may experience psychological vulnerability, as they are exposed to a dual burden: being expected to achieve academic success while simultaneously assisting their families, often without sufficient academic support from their mothers because of language barriers. To mitigate these challenges, it is essential to not only enhance marriage immigrant mothers’ understanding of the host country’s educational system but also establish structural and ongoing support for career exploration and academic development among adolescents from marriage immigrant families. Such interventions may help reduce the risk of mental health issues caused by academic stress [36], alleviate feelings of guilt experienced by mothers who are unable to provide adequate academic support to their children [37], and ease the burden on native fathers who assume additional familial roles [38].

Finally, the participants expressed emotional distress stemming from communication failures with their immigrant mothers. Repeated breakdowns in communication—due to language barriers and emotionally closed interactions—led some participants to give up trying to communicate their emotions and even to develop negative perceptions of their mothers’ foreign identities. Immigrant mothers often have difficulty expressing subtle emotions in Korean [39] and tend to engage in surface-level conversations with their spouses, lacking emotional depth [40]. Furthermore, the cultural norms of emotional restraint in Asian families [14,41] discourage adolescents from openly expressing negative emotions to their parents. However, the quality of parent–child communication is a critical factor affecting adolescent mental health, including depression, anxiety, and suicidal ideation [42]. Therefore, family-based programs that promote open communication and enhance immigrant mothers’ open and emotional communication skills are urgently needed to support the well-being of adolescents in marriage immigrant families.

## 5. Conclusions

This study employed IPA to explore and deeply understand the family caregiving experiences and their significance among adolescents from marriage immigrant families. Through this analysis, three superordinate themes were identified namely, “Old enough to help—naturally, and rightly so”, “Foreign mother whom I naturally come to help”, and “Unavoidable family assistance, even under pressure”. Additionally, 10 subordinate themes were identified.

Although this study was initially designed to investigate experiences of parentification among adolescents in marriage immigrant families, the research question being partially revised during the course of the in-depth interviews and data analysis has presented a limitation. The original design anticipated that adolescents in marriage immigrant families would take on additional responsibilities due to their immigrant parent’s background and that these roles might involve a certain level of burden. However, the findings revealed that such roles were predominantly undertaken by the native Korean fathers, who were adults and citizens of Korea, rather than by the minor children who were born and raised in the country. Consequently, the typical indicators of parent–child role reversal or high levels of responsibility assumed by the child were not clearly observed. This necessitated iterative revisions to the research question, which may be considered a limitation of this study.

Additionally, some of this study’s participants were recruited through an online community primarily composed of Filipino marriage immigrant women working as English teachers in Korea. This recruitment channel implies that the participants’ mothers generally had a relatively high level of education, which may pose certain limitations to the generalizability of the findings.

Lastly, all the participants in this study were adolescents from families with immigrant mothers and native Korean fathers, despite the absence of such restrictions in the recruitment process. This may reflect broader demographic patterns in South Korea, where the majority of marriage-based immigration involves immigrant women marrying Korean men; in fact, such unions account for 81.8% of all international marriages in the country [43]. While this limits the applicability of the findings to families with immigrant fathers, the results nonetheless offer valuable insights into the dominant family structure among multicultural families in Korean society.

Based on the findings and limitations of this study, the following directions for future research are proposed. First, further qualitative studies should explore the nature and psychological impact of family assistance among adolescents in families in which the native Korean parent is absent or plays a minimal role. Second, quantitative research is needed to assess the psychological distress experienced by immigrant spouses and adolescent children in contexts where the role of the native parent is diminished. Finally, intervention programs should be developed and empirically tested to address the communication challenges and lack of open dialogue between marriage immigrant mothers and their children, as identified in this study.

## Figures and Tables

**Table 1:** Characteristics of the participants.

Participant No.		1	2	3	4	5	6	7	8	9	10
Age		13	17	17	18	13	14	14	14	13	17
Sex		Female	Male	Female	Male	Female	Female	Female	Male	Female	Female
Age of Father		45	50	51	61	63	56	54	57	47	52
Age of Mother		34	42	41	40	40	34	35	35	35	55
Father’s Educational Level		High school	Under-graduate	Middle school	Under-graduate	High school	High school	High school	High school	High school	High school
Mother’s Educational Level		Middle school	Under-graduate	Under-graduate	Graduate	Under-graduate	Primary school	Under-graduate	High school	High school	Under-graduate
Mother’s Country of Origin		Pakistan	Philippines	Philippines	Philippines	Philippines	Vietnam	Vietnam	Vietnam	Vietnam	Indonesia
Mother’s Korean Proficiency	Speaking	High	High	High	Low	High	Middle	High	High	High	High
	Listening	High	Middle	High	Middle	High	Middle	High	High	Middle	High
	Reading	Middle	Middle	Middle	Middle	High	Low	High	Middle	Middle	Middle
	Writing	Middle	Middle	Middle	Low	Low	Low	High	High	Low	Middle
Age of Sibling(s)		11, 5				32, 29	13	12	3		18

**Table 2:** List of themes and subthemes of the family assistance experiences of adolescents in marriage immigrant families.

Theme 1. Old enough to help—naturally, and rightly so (1) Things that an adolescent—not a child—can take on (2) Learning and preparing for the tasks I will face when I grow up (3) Family means helping one another.
Theme 2. Foreign mother whom I naturally come to help (1) Being told to help my immigrant mother (2) Driven by empathy for my immigrant mom (3) Assisting with mom’s small but constant language struggles
Theme 3. Unavoidable family assistance, even under pressure (1) Carrying the weight of the father’s role too soon (2) Forced into traditional but outdated roles (3) Unassisted academic struggles (4) A language barrier too thick to truly touch the heart

## Data Availability

The data generated and analyzed in this study are not publicly accessible due to the sensitive and personal nature of the information shared by vulnerable participants.

## Author’s Note

This article is based on the first author’s doctoral dissertation submitted to Seoul National University in 2023, with revisions and enhancements made for publication.
